# Multiple Functional Risk Variants in a SMAD7 Enhancer Implicate a Colorectal Cancer Risk Haplotype

**DOI:** 10.1371/journal.pone.0111914

**Published:** 2014-11-06

**Authors:** Barbara K. Fortini, Stephanie Tring, Sarah J. Plummer, Christopher K. Edlund, Victor Moreno, Robert S. Bresalier, Elizabeth L. Barry, Timothy R. Church, Jane C. Figueiredo, Graham Casey

**Affiliations:** 1 Department of Preventive Medicine, University of Southern California, Los Angeles, California, United States of America; 2 Cancer Prevention and Control Program, Catalan Institute of Oncology, CIBERESP and University of Barcelona, Hospitalet de Llobregat, Barcelona, Spain; 3 Department of Gastroenterology, Hepatology and Nutrition, University of Texas MD Anderson Cancer Center, Houston, Texas, United States of America; 4 Department of Community and Family Medicine, Geisel School of Medicine at Dartmouth, Lebanon, New Hampshire, United States of America; 5 Division of Environmental Health Sciences, University of Minnesota School of Public Health, Minneapolis, Minnesota, United States of America; National Cancer Institute, National Institutes of Health, United States of America

## Abstract

Genome-wide association studies (GWAS) of colorectal cancer (CRC) have led to the identification of a number of common variants associated with modest risk. Several risk variants map within the vicinity of TGFβ/BMP signaling pathway genes, including rs4939827 within an intron of *SMAD7* at 18q21.1. A previous study implicated a novel SNP (novel 1 or rs58920878) as a functional variant within an enhancer element in *SMAD7* intron 4. In this study, we show that four SNPs including novel 1 (rs6507874, rs6507875, rs8085824, and rs58920878) in linkage disequilibrium (LD) with the index SNP rs4939827 demonstrate allele-specific enhancer effects in a large, multi-component enhancer of *SMAD7*. All four SNPs demonstrate allele-specific protein binding to nuclear extracts of CRC cell lines. Furthermore, some of the risk-associated alleles correlate with increased expression of *SMAD7* in normal colon tissues. Finally, we show that the enhancer is responsive to BMP4 stimulation. Taken together, we propose that the associated CRC risk at 18q21.1 is due to four functional variants that regulate *SMAD7* expression and potentially perturb a BMP negative feedback loop in TGFβ/BMP signaling pathways.

## Introduction

TGFβ signaling has long been associated with colorectal cancer (CRC). In addition to canonical roles in the regulation of apoptosis, cell differentiation, and cell growth of intestinal epithelium, TGFβ signaling is an important player in the immune response and inflammatory bowel disease, a risk factor for CRC (reviews [1]–[3]). TGFβ and BMP signaling define the two major branches of the TGFβ pathway. Activation of either branch leads to the recruitment of R-SMADs, SMAD2/3 in the case of TGFβ or SMAD1/5/8 in the case of BMPs, which form a complex with SMAD4. This complex then directs the transcription of many target genes, including *SMAD7*. SMAD7, an inhibitory SMAD like SMAD6, in turn serves as a negative feedback regulator of TGFβ and BMP signaling, in addition to acting as a crosstalk node with other pathways including TNF [4], [5].

SMAD7 also plays other important roles in the etiology of CRC, such as interacting with β-catenin to regulate *MYC* expression and WNT signaling [6]. *SMAD7* overexpression has been seen in some CRC cells, and reduction of *SMAD7* expression using anti-sense RNA leads to decreased proliferation in the HCT-116 CRC cell line and human CRC neoplastic explants, and reduced tumorigenesis in the APC^min/-^ mouse [7].

CRC GWAS have led to the identification of several genomic regions associated with risk that include genes in the TGFβ signaling pathway including *SMAD7*, *BMP2*, *BMP4*, and *GREM1* [8]–[14]. This includes single nucleotide polymorphism (SNP) rs4939827, located in *SMAD7* intron 4, which has been reported in multiple studies. Pittman et al. reported that a novel SNP (termed novel 1, later renamed rs58920878) mapped to an enhancer element that drove GFP expression in *Xenopus* tadpole muscles and colorectum in an allele specific manner, implicating rs58920878 as a functional variant within this region [15].

Prompted by our discovery of multiple functional variants within the 11q23 CRC GWAS region [16], we comprehensively examined the genomic region surrounding the CRC tagSNP rs4939827 at 18q21.1 for other potential functional SNPs. We identified 4 SNPs, rs6507874, rs6507875, rs8085824 and rs58920878 (novel 1), in a region of approximately 2 kb, each demonstrating allele-specific enhancer activity. We further determined that alleles corresponding to the risk haplotype correlated with increased expression of *SMAD7* in normal colon epithelial tissues. Sequences encompassing all four SNPs also bound to nuclear proteins from CRC cell lines in an allele-specific manner in electrophoretic mobility shift assays (EMSAs). Finally, we showed that the enhancer was responsive to BMP4-induced signaling in luciferase assays, while neither haplotype was responsive to TGFβ1. Taken together, we propose that the CRC risk at chromosome 18q21.1 is due to the contributions of 4 functional variants in an enhancer affecting the expression of *SMAD7*, potentially leading to perturbed regulation of the BMP negative feedback loop in BMP/TGFβ signaling pathways.

## Results

### Region Analysis

**Figure 1:**
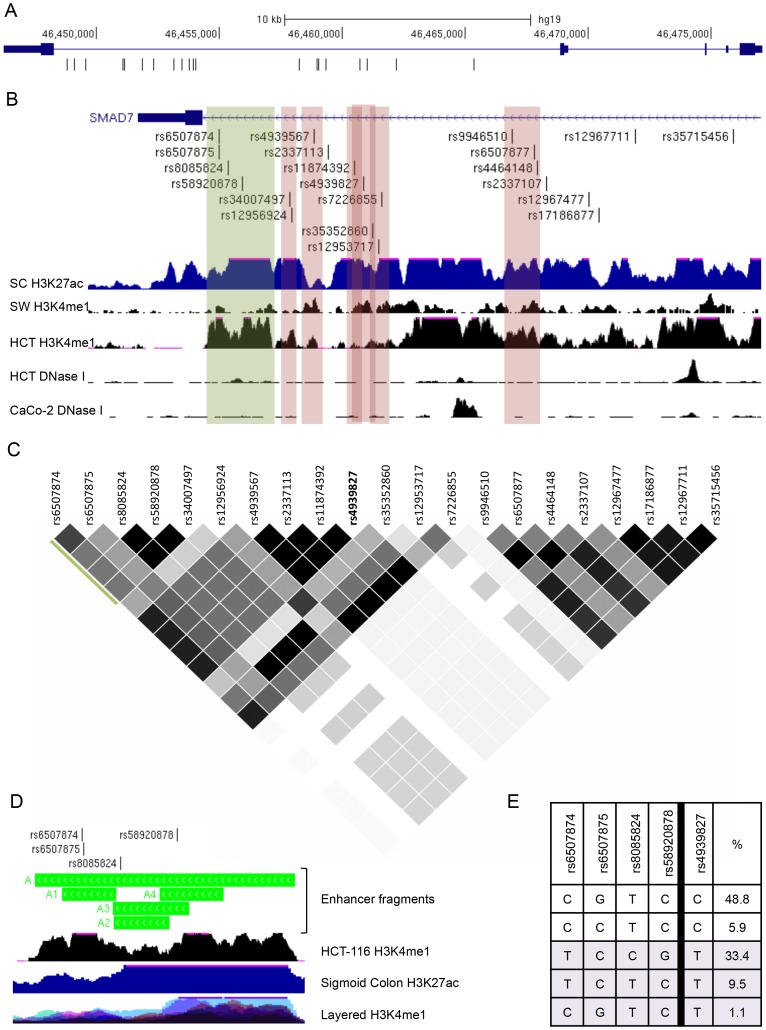
Chromatin features and LD structure for the 18q21.1 CRC GWAS locus. (**A**) The *SMAD7* gene depicted with genomic coordinates (hg19) and the position of SNPs in LD (r^2^≥0.2, CEU population) with tagSNP rs4939827. (**B**) UCSC Genome Browser view of SMAD7 with SNPs in LD with rs4939827 indicated. ChIP-seq tracks for enhancer histone marks are Sigmoid Colon (SC) H3K27ac from REMC/UCSD for the Roadmap Epigenomics Consortium, SW480 (SW) H3K4me1, and HCT-116 (HCT) H3K4me1 from the ENCODE consortium. DNase I hypersensitivity tracks HCT-116 (HCT) and CaCo-2 below are from the UW ENCODE dataset. The green stripe represents the enhancer fragment A. Red stripes indicate cloned fragments B to G (left to right) that were shown to lack enhancer activity. Coordinates for each fragment are provided in File S1. (**C**) Linkage disequilibrium plot for rs4939827 including all SNPs in 1KG project with r^2^≥0.2, created with Haploview. r^2^ = 1- black, 1>r^2^>0.2 - grey scale. (**D**) Zoomed view of fragment A showing sub-fragments A1–A4. The ENCODE Layered H3K4me1 track of 7 cell lines is shown to identify cell-type specific peaks. (**E**) Haplotypes and percentages (CEU population) for the 4 SNPs in fragment A and rs4939827. Haplotypes associated with the rs4939827 risk allele T are in light purple.

The index SNP rs4939827 associated with CRC at chromosome 18q21.1 lies within intron 4 of the *SMAD7* gene. There are 20 SNPs in LD with rs4939827 with an r^2^≥0.2 in the CEU population (1000 Genomes Project, June 2011 release), our selected LD threshold for potential functional candidates (Figure 1A and 1C). All 21 SNPs lie within a 16 kb region of the *SMAD7* intron 4. In order to identify potential genomic regulatory sequences from this region, the SNPs in LD with rs4939827 were aligned with chromatin immunoprecipitation and sequencing (ChIP-seq) tracks for histone methylation and acetylation marks associated with enhancers, H3K4me1 and H3K27ac (Figure 1B). For this study, we referenced Sigmoid Colon H3K27 acetylation from the Roadmap Epigenomics Consortium [17], and CRC cell lines SW480 and HCT-116 H3K4 monomethylation generated in our laboratory and from the ENCODE project, respectively [16], [18], [19]. Several potential enhancer peaks containing SNPs in LD with rs4939827 were identified in sigmoid colon, SW480, or HCT-116 cells, including a 2kb region containing rs58920878 (green stripe, Figure 1B). In order to characterize the region comprehensively, smaller peaks below the threshold for peak calling software were also cloned and tested for activity if they contained a SNP meeting our LD cut-off. DNase I hypersenstitivity tracks from the ENCODE project were also aligned, but no peaks overlapped with any of the candidate SNPs in LD with rs4939827 [20], [21].

### Enhancer Activity Assays

**Figure 2:**
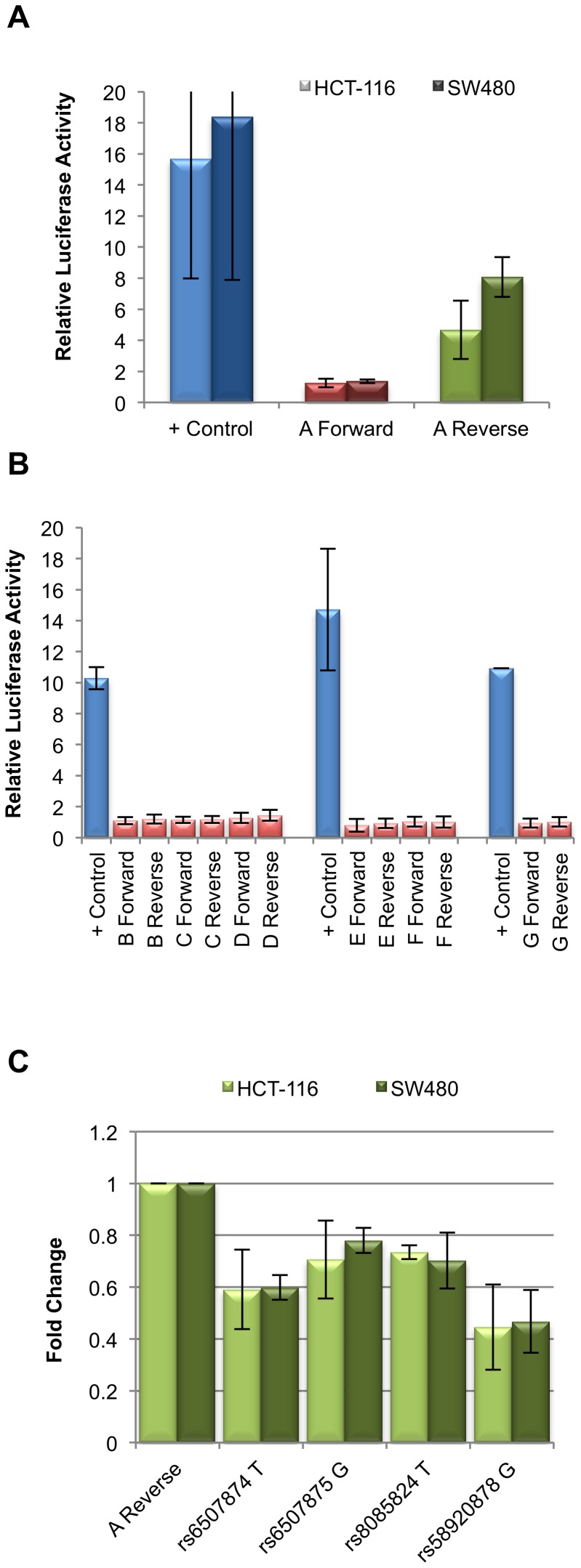
Fragment A exhibits enhancer activity in the reverse orientation only. (**A**) Relative luciferase activity for positive control, fragment A in the forward, and fragment A in the reverse orientation. Light columns indicate activity in HCT-116, dark columns indicate activity in SW480. (**B**) Fragments B through G, shown in figure 1B as red stripes do not show enhancer activity in either orientation in HCT-116. Positive controls are shown for each group of constructs analyzed on the same plate. Fragment E contains the tagSNP rs4939827. (**C**) Activity of fragment A in the reverse orientation with all SNPs in the fragment with their C allele, with each allele changed independently to the alternate allele, shown as fold change relative to the construct containing the four C alleles. All four alleles result in a statistically significant reduction in activity: rs6507874 T *p* = 8.25×10^−3^ (HTC-116), *p* = 3.30×10^−2^ (SW480); rs6507875 G *p* = 3.40×10^−2^ (HCT-116), *p = *6.79×10^−3^ (SW480); rs8085824 T *p = *1.20×10^−2^ (HCT-116), *p = *3.12×10^−3^ (SW480); rs58920878 G *p = *2.09×10^−3^ (HCT-116), *p = *3.05×10^−3^ (SW480).

Seven putative enhancer regions were cloned into a luciferase assay vector to determine enhancer activity in CRC cell lines HCT-116 and SW480. Only the 2 kb fragment (green stripe in Figure 1B) at the 3′ end of *SMAD7* intron 4 showed activity in our assays, but only in the reverse orientation (Figure 2A). Regions tested but lacking activity in this assay are indicated in red stripes on Figure 1B (Figure 2B). The 2 kb enhancer region, we term fragment A, contains 4 SNPs in LD with rs4939827: rs6507874, rs6507875, rs8085824, and rs58920878 (novel 1). An expanded view of fragment A is shown in Figure 1D. As shown in Figure 1C, these four SNPs are in LD (r^2^ = 1 to 0.494) with one another and define 5 haplotypes in the CEU population. These haplotypes are shown in relation to the rs4939827 variant (risk allele T) in Figure 1E [22], [23].

We first tested whether there were any allele-specific differences in enhancer activity for each of the SNPs individually starting with a 2 kb fragment with all 4 SNPs containing C alleles. While this haplotype is not seen in the CEU population, this allele combination showed the highest enhancer activity of all fragments tested. We observed allele-specific changes in enhancer activity for each of the four variants when alleles were changed by site directed mutagenesis (Figure 2C). All 4 SNPs showed a decrease in enhancer activity when alleles were changed to the alternate form. The largest decrease in activity was seen when rs58920878 was changed from C to G. While the minor alleles for SNPs rs6507874 (T, minor allele frequency (MAF)  = 0.44) and rs58920878 (G, MAF  = 0.34) showed a lower enhancer activity, the minor alleles for SNP rs8085824 (C, MAF  = 0.34) and rs6507875 (C, MAF  = 0.49) showed a higher enhancer activity in these assays.

**Figure 3:**
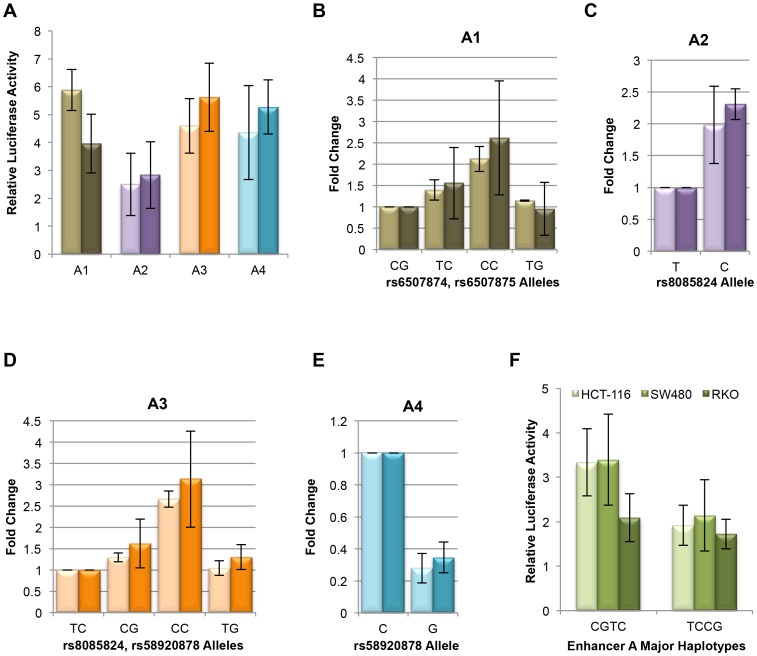
Fragment A is composed of smaller domains with independent enhancer activity. (**A**) Fragment A was divided into 4 smaller DNA fragments, A1–A4, depicted in Figure 1D. Each smaller fragment demonstrated enhancer activity in both HCT-116 (light bars) and SW480 (dark bars). For the experiment shown, all SNPs contained their C alleles. (**B**) Activity of Fragment A1 containing rs6507874 and rs6507875, shown as fold change relative to the major haplotype CG for HCT-116 (light bars) and SW480 (dark bars). The minor haplotype TC (*p* = 2.25×10^−2^ (HCT-116), *p* = 8.59×10^−2^ (SW480)), and combination CC (*p* = 5.06×10^−5^ (HCT-116), *p* = 1.75×10^−5^ (SW480)) not seen in the CEU population show higher activity than the major haplotype. Combination TG (*p* = 0.450 (HCT-116), *p* = 0.287 (SW480)) demonstrates activity similar to CG. (**C**) Fragment A2 containing rs8085824 shows 2-fold higher enhancer activity with the minor C allele of than the major allele T (*p = *4.75×10^−3^ (HCT-116), *p = *3.61×10^−4^ (SW480)). (**D**) Activity of fragment A3 containing rs8085824 and rs58920878 relative to major haplotype TC. The minor haplotype CG demonstrates higher activity than TC (*p = *2.49×10^−2^ (HCT-116), *p = *6.32×10^−3^ (SW480)). The T (major) allele of rs8085824 shows lower activity than C (minor) allele regardless of rs58920878 allele. The highest activity is seen in haplotype CC that is not reported in the CEU population (*p = *6.35×10^−7^ (HCT-116), *p = *1.47×10^−6^ (SW480)). (**E**) Activity of fragment A4 encompasses the peak containing rs58920878. The minor allele G of rs58920878 shows dramatically less activity than the major C allele (*p = *1.92×10^−4^ (HCT-116), *p = *1.52×10^−5^ (SW480)). **(F)** Fragment A was tested for activity with the two most common haplotypes (Figure 1E) in the CEU population. The CGTC haplotype shows higher activity in HCT-116 (light bars, *p* = 1.04×10^−11^) and SW480 (medium bars, *p* = 8.60×10^−11^), but no significant difference in activity in RKO (dark bars).

Fragment A encompasses several smaller distinct HCT-116 H3K4me1 peaks, with the left-most peak specific to HCT-116 when compared to the layered H3K4me1 super-track of 7 ENCODE Tier 1 cell lines [19] (Figure 1D). To test the relative contributions of these regions and SNPs individually on overall enhancer activity, 4 smaller constructs, A1–A4, were designed (Figure 1D). The A1 fragment includes SNPs rs6507874 and rs6507875 and encompasses the HCT-116 specific peak. These two SNPs are only separated by 13 bp, and are in LD with one another with r^2^ = 0.794, D′ = 1. As shown in Figure 3A, the A1 fragment demonstrated enhancer activity. The major haplotype of rs6507874 and rs6507875 alleles in the CEU population, CG, demonstrated the lowest level of enhancer activity compared to the other three possible allele combinations (Figure 3B). The TC and CC minor haplotypes showed approximately 1.3–1.5 fold and 2–2.5 fold higher activity in both cell lines, HCT-116 and SW480, respectively (Figure 3B). The TG allele combination, while not a known CEU haplotype, showed lower activity than the TC haplotype. Taken together, we conclude that while both SNPs, rs6507874 and rs6507875, contribute to the overall enhancer activity level of the A1 fragment, rs6507875 alleles show a larger allele-specific effect than rs6507874, in the direction consistent with the 2 kb fragment (Figure 2C). In contrast, the effect of the rs6507874 allele on enhancer activity was dependent upon which rs6507875 allele was present, suggesting a contextual effect within the enhancer components. These data imply a complex functional relationship exists between adjacent SNPs.

The A2 fragment contains SNP rs8085824 and straddles the region between the two most prominent HCT-116 H3K4me1 peaks in enhancer A (Figure 1D). While fragment A2 had activity on its own, it showed the lowest activity of the 4 sub-regions tested (Figure 3A). When the rs8085824 allele was changed from T (major allele) to C (minor allele), activity increased two fold (Figure 3C) consistent with results for the larger fragment (Figure 2C). The A3 fragment encompasses the A2 region (including rs8085824) and a further 160 bp to include rs58920878. This fragment showed 2 fold more activity than the A2 fragment. The major haplotype of rs8085824 and rs58920878 (r^2^ = 1, D′ = 1, CEU) is TC. The minor haplotype CG demonstrated 1.3 to 1.6 fold higher activity than the major haplotype in HTC-116 and SW480 cells, respectively. The artificially constructed CC allele combination had the highest level of activity tested for the A3 fragment. Comparing the CC fragment to the TC and CG haplotype fragments, we observed that a larger drop in activity resulted from changing the rs8085824 allele than rs58920878. Conversely, changing rs58920878 in the TC haplotype to TG did not result in a significant difference in enhancer activity. As with the A2 fragment, for SNP rs8085824 these results were consistent with allele-specific effects seen using the larger fragment (Figure 2C). However, for SNP rs58920878, the effect predicted by the 2 kb fragment was dependent upon which rs8085824 allele was present, again, suggesting that a complex functional relationship exists between adjacent SNPs.

Finally, fragment A4, encompassing the second smaller HCT-116 H3K4me1 peak, contained only SNP rs58920878 and demonstrated similar activity to fragments A1 and A3 (Figure 3A). This fragment showed enhancer activity was reduced 3 fold when the major allele C was changed to the minor allele G (Figure 3E). The A4 fragment containing rs58920878 was the only one of the smaller fragments where the minor allele or haplotype demonstrated lower activity than the major allele/haplotype.

The two most common haplotypes in CEU for this group of 4 SNPs are CGTC (49.9%) and TCCG (33.4%) (Figure 1E). These combinations were tested in the context of the 2 kb enhancer A construct. As shown in Figure 3F, overall the major CGTC haplotype demonstrated higher enhancer activity than the minor TCCG haplotype in HCT-116 and SW480. Interestingly, when we tested the two haplotypes in the CRC cell line RKO, CGTC was not significantly more active than the TCCG haplotype, indicating there was some specificity to fragment A activity even amongst CRC cell lines. Neither haplotype of fragment A was active in non-CRC cell line HEK293 that served as a negative control (Figure S1A). Fragments containing haplotypes TCTC (9.5% of CEU population) and CCTC (5.9% of CEU population) demonstrated activity levels between that of haplotypes CGTC and TCCG (Figure S1B).

### Electrophoretic Mobility Shift Assays

**Figure 4:**
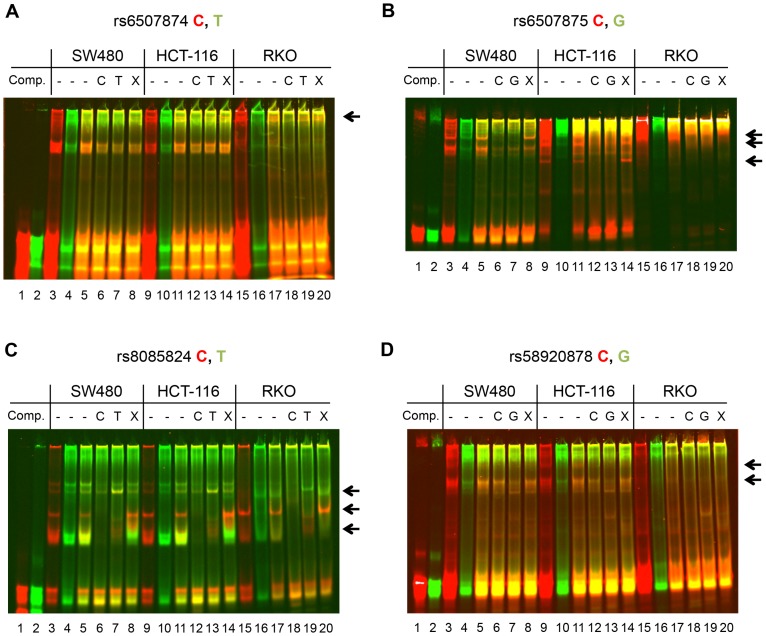
Differential protein binding by SNP alleles using EMSA. (**A**) Nuclear extracts from SW480, HCT-116, and RKO cell lines were incubated with IR-dye labeled 33mers centered on rs6507874 C (red label) and T (green labels) prior to native EMSA. Lanes 1 and 2 show probe without nuclear extract. Lanes 3, 4, 9, 10, 15, and 16 show each labeled probe binding to nuclear proteins individually with cell line as noted above. Lanes 5–8, 11–14, and 17–20 are a 1∶1 competition with labeled C and T allele probes. Lanes 6, 12 and 18 contain 200-fold excess unlabeled C allele competitor. Lanes 7, 13, and 19 contain 200-fold excess unlabeled T allele competitor. Lanes 8, 14, and 20 contain 200-fold excess competitor of an unmatching sequence with similar nucleotide content. Bands specific for one allele and lost upon competition are marked with arrows. See Figure S2 for red (700) channel image of the C probe and the green (800) channel of the T probe in black and white. (**B**) As in panel A, with rs6507875 C allele (red probe) and G allele (green). See Figure S3 for red (700) channel image of the C probe and the green (800) channel of the G probe in black and white. (**C**) As in panel A, with rs8085824 C allele (red) and T allele (green). See Figure S4 for red (700) channel image of the C probe and the green (800) channel of the T probe in black and white. (**D**) As in panel A, with rs58920878 C allele (red) and G allele (green). See Figure S5 for red (700) channel image of the C probe and the green (800) channel of the G probe in black and white.

To better understand these allele-specific enhancer effects, we next investigated the nuclear protein binding to the sequences containing each of the 4 SNPs, rs6507874, rs6507875, rs8085824, and rs58920878. We hypothesized that transcription factors would selectively bind to the C alleles with higher affinity, as the 2 kb fragment and fragments A1–A4 with C alleles showed the highest level of enhancer activity. Conversely, inhibitory proteins may bind to the T or G alleles preferentially to negatively regulate enhancer activity. To this end, 33 bp double stranded oligonucleotides centered on each SNP, were synthesized. In each case, the C allele was labeled with the red (700) IR dye. The alternate alleles, either T or G were labeled with green (800) IR dye (individual channels are presented as black and white images in Figures S2–S5 for ease of reproduction and analysis). Nuclear extracts were prepared from SW480, HCT-116 and RKO CRC cell lines and incubated with non-specific and specific unlabeled competitor DNA as indicated in Figure 4. The two alleles of each SNP are labeled with different colors, allowing a direct comparison of the binding of each allele to nuclear proteins in the same reaction. As shown in Figure 4, for each probe set tested, there are bands specific to one allele versus the other allele.

For each allele set, lanes 3, 4, 9, 10, 15 and 16 show a single allele probe incubated with the nuclear extract. Lanes 5–8, 11–14, and 17–20 contain reactions with 1∶1 amounts of each labeled allele. To determine specificity of the shifted complexes, unlabeled competitive oligonucleotides for each allele were added in 200-fold excess. Lanes 6, 12, and 18 contain the unlabeled C alleles and lanes 7, 13 and 19 contain the unlabeled T or G alleles. In lanes 8, 14, and 20, an unlabeled competitor of similar base composition but not matching any of the sequences was added in an equal amount. Bands that disappeared when competed with either SNP allele, but present with the unrelated (non-specific) competitor were considered specific for the SNP sequence. The most prominent of the specific bands are marked in the margins with arrows.

In the case of rs6507874 and rs58920878 (Figure 4A, 4D), there were complexes found in HCT-116 and SW480, which were not seen in RKO. This may explain why haplotype specific activity was not seen in the RKO luciferase activity experiment. In Figures 4A and S2, the band marked by the arrow was specific to the rs6507874 C allele probe. Note that in HCT-116 and RKO extracts, even 200-fold excess of the unlabeled T allele oligonucleotide could not out-compete the C allele for binding. For the rs6507875 probes (Figure 4B and S3), while there were specific bands for both alleles (arrows), the G allele bound with more affinity than the C allele. Interestingly, rs6507875 probes bound strongly to components of the RKO extract, even in the presence of excess unlabeled DNA (Figure 4B).

For the rs8085824 probes, we again found several specific complexes for each allele. One prominent complex, marked by the bottom arrow, showed that the C allele probe was not completely outcompeted with 200-fold excess of the T allele. It appears that the bound proteins of this complex are found in higher amounts in SW480 and HCT-116 than RKO nuclear extracts. In the case of the rs58920878 probes, we found several bands specific for the C allele, while the G allele was found predominantly in larger complexes (higher bands) than the C allele.

Determining the identity of these allele-specific binding proteins will be required to fully understand the action of the SMAD7 enhancer. Protein binding prediction software Biobase Match, based on the TRANSFAC motif database, was used to identify candidates for each SNP region [24]. Results of this analysis are presented as Tables S1, S2 and S3 in File S1. The DNA binding protein with the top difference in predicted binding scores between alleles for rs6507874/rs6507875 (analyzed together due to proximity) was NF-1A, for rs8085824 was Churchill, and for rs58920878 was ZF5. However, for each locus, none of the proteins with the greatest predicted allele differences were the proteins with the highest core or matrix scores for each sequence.

### eQTL Analysis in Normal Colon Tissue

We next asked if the genotype at these SNPs correlated with gene expression levels in normal colon tissues. As the enhancer is located in intron 4 of the *SMAD7* gene, *SMAD7* was an obvious candidate target gene. In addition to rs6507874, rs6507875 and rs8085824, we also genotyped the tagSNP rs4939827 in pathologically normal human tissue samples obtained from surveillance colonoscopy through the Aspirin/Folate Polyp Prevention Study [25]–[27]. We were unable to design a TaqMan assay for rs58920878, however SNPs rs8085824 and rs58920878 are in perfect LD (r^2^ = 1, D′ = 1); therefore rs8085824 also represents a proxy for rs58920878. No statistically significant correlation between expression of the two other genes within 0.5 Mb of rs4939827, *CTIF* or *DYM*, and any of the genotyped SNPs was seen (data not shown).

**Figure 5:**
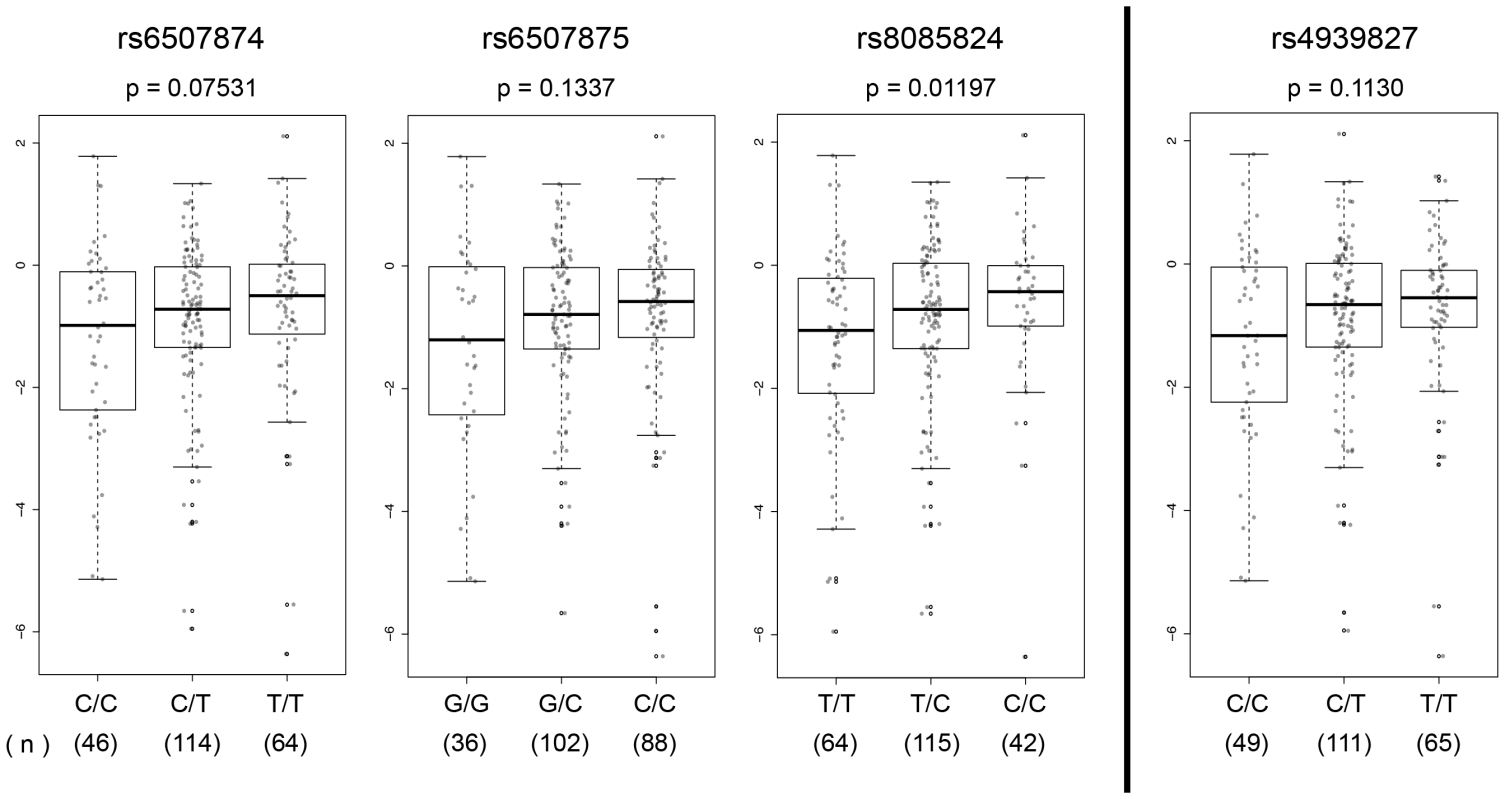
Candidate functional SNPs are association with *SMAD7* gene expression levels. Fold change (FC) of SMAD7 expression was measured in normal colon tissue samples from surveillance colonoscopies. Rank-based non-parametric analysis was used to estimate the effect of each extra minor allele for rs6507874, rs6507875, rs8085824, and rs4939827 (additive model) on gene expression adjusting for gender, age and race. Two-sided *p*-values were obtained from a likelihood ratio test. Log_2_(ΔΔCt) were plotted as a function of genotypes using a box plot – dot plot overlay. Number of samples is noted under each genotype. A statistically significant association was found for rs8085824.

We found that the tagSNP rs4939827 did not show a statistically significant association with *SMAD7* expression levels, although higher expression trended with the T GWAS risk allele (p = 0.1130) (Figure 5). However, SNP rs8085824 C (minor) allele demonstrated a statistically significant correlation (*p* = 0.01197) with increased *SMAD7* expression. Given that SNPs rs8085824 and rs58920878 are in perfect LD, this result can be extrapolated to imply a correlation between rs58920878 G (minor) allele and higher levels of *SMAD7* expression. The rs6507874 T (minor) allele (p = 0.07531) and the rs6507875 C (minor) allele (*p* = 0.1337) did not show a statistically significant correlation with increased *SMAD7* expression. Note that overall the TCCG haplotype (minor haplotype) correlated with higher *SMAD7* expression in tissues and in luciferase assays in the context of fragments A1, A2, and A3, but was opposite to the effect seen for enhancer activity of the 2 kb fragment in luciferase experiments where the fragment containing the TCCG haplotype (minor haplotype) correlated with reduced enhancer activity compared to the fragment containing the CGTC haplotype.

Further studies will be needed to determine how this multi-component enhancer controls *SMAD7* gene expression during colon development and cell growth, and to determine whether or not the individual components of the enhancer act independently of one another.

### Regulation of the SMAD7 Enhancer

**Figure 6:**
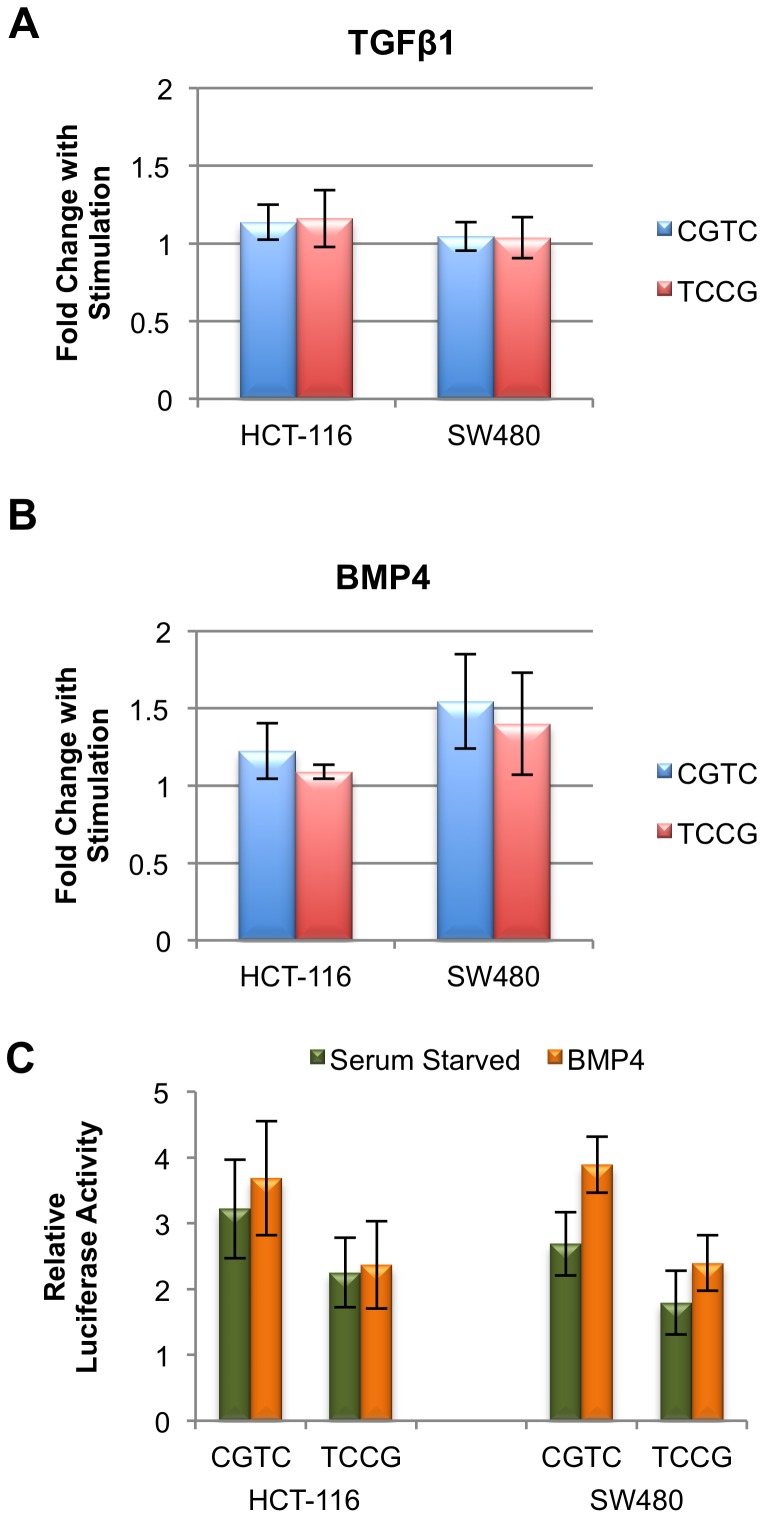
Fragment A Enhancer is responsive to BMP4 stimulation but not TGFβ1. (**A**) Enhancer activity was measured by luciferase assay for the fragment A containing the major (CGTC) and minor (TCCG) haplotypes in serum starved HCT-116 and SW480 cells incubated with TGFβ1 for 6 hours. Samples are plotted as a fold change in activity relative to non-treated cells on the same plate. (**B**) Enhancer activity was stimulated by 6 hours of BMP4 treatment for the CGTC haplotype in HCT-116 (*p* = 4.14×10^−3^) and SW480 cells (*p* = 1.22×10^−10^). The TCCG haplotype in fragment A shows lower levels of stimulation, and only in SW480 (*p* = 1.61×10^−6^). (HCT-116 *p* = 0.38). Effect of BMP is plotted as fold change over untreated samples for each haplotype on the same plate. (**C**) Enhancer activity comparison between the CGTC and TCCG haplotypes following BMP4 treatment. Relative luciferase activity was plotted for each haplotype in HCT-116 and SW480 using the same experimental dataset as (B).

We next wanted to determine the relationship between enhancer activity and BMP/TGFβ signaling. As mentioned in the introduction, SMAD7 acts as a key mediator of the negative feedback loop for both the TGFβ and BMP signaling pathways. We were interested therefore to determine if enhancer A was a part of a feedback loop, becoming more active in response to either TGFβ1 or BMP4 signaling. HCT-116 and SW480 cells were serum starved overnight prior to 6 hours of treatment with 100 pmol TGFβ1 or BMP4, and the 2 kb fragment A was used to test for luciferase activity. Following treatment with TGFβ1, fragments containing either the CGTC (major) haplotype or the TCCG (minor) haplotype demonstrated no statistically significant change in activity in either cell line (Figure 6A). In contrast, following treatment with BMP4, enhancer activity of the fragment containing the CGTC major haplotype was increased 1.2 fold in HCT-116 cells and 1.5 fold in SW480 cells (Figure 6B). An increase in enhancer activity was also seen with the fragment containing the TCCG minor haplotype in SW480 cells where BMP treatment led to a 1.4 fold increase in enhancer activity, but not in HCT-116 cells where no significant increase was observed (*p* = 0.38) (Figure 6B). Because DNA fragments containing both haplotypes were stimulated by BMP4 in SW480 to a similar extent (fold change), we postulate that the 4 SNPs in the risk haplotype do not disrupt the actual BMP responsive components of the enhancer. However, due to the deficit in activity with the minor haplotype, following BMP4 stimulation, the TCCG haplotype construct still demonstrates lower enhancer activity than the CGTC haplotype without BMP4 stimulation (Figure 6C). In total, these data implicate the enhancer in a negative feedback loop in response to BMP signaling but not in the TGFβ arm of the signaling cascade.

## Discussion

Recently, our lab and others have begun to consider the effects of multiple functional variants in linkage disequilibrium (LD) contributing to disease risk identified through GWAS instead of a single functional variant [28]. For example, at chromosome 11q23.1, our lab identified two SNPs in LD (r^2^ = 1) associated with CRC risk, one in an enhancer and one in a bi-directional promoter, that demonstrated allele-specific activity and correlated with expression levels of three previously uncharacterized gene targets [16]. Furthermore, recent advances in understanding the biology of enhancers, specifically multi-component, super or stretch enhancers prompted us to take a deeper look at 18q21.1 that contains several histone-modification ChIP-seq peaks associated with active enhancers mapping to *SMAD7* intron 4 [29]–[31].

SMAD7 functions as an important pathway regulation step in the signaling of the TGFβ superfamily. SMAD7 is also a nexus of crosstalk for other key signaling networks, including WNT and TNF. Thus, it is not surprising that SMAD7 levels are exquisitely controlled in tissues given the importance of TGFβ/BMP signaling particularly in colon cells. This control is mediated, in part, at the gene expression level by multiple enhancer elements that bind transcription factors and co-activators to respond to specific cellular conditions and signaling programs. Most studies characterizing the enhancers that control *SMAD7* have been performed in mouse [32]. BMP and TGFβ response elements have been identified in the promoter region, and an enhancer in intron 1 was discovered to have BMP responsiveness dependent on GATA-family transcription factors [33].

Hnisz et al. recently classified nearly the entire length of the human *SMAD7* gene as a super-enhancer in colon crypts, sigmoid colon and fetal intestine [29]. Super-enhancers are defined as large enhancer clusters that define a cell's identity [29], [31]. The 2 kb enhancer element described in this work is only one segment of this super-enhancer. Within the 2 kb region, we found smaller components, each with enhancer activity. We showed that the activity of these four smaller enhancer elements was modulated by common SNPs in LD with the CRC risk tagSNP rs4939827. Each SNP contributed to the overall level of activity of the enhancer by binding nuclear proteins, presumably transcription factors, in an allele-specific manner. How these individual SNP's effects are manifest in the colon tissue of an individual is unclear. EMSA analysis showed unique binding patterns for each sequence surrounding the SNPs, meaning that each enhancer segment could be responsive to different cell stimuli. That we did not always observe a consistent relationship between the alleles of the 4 SNPs under study in both their effect on enhancer activity as well as their correlation with *SMAD7* gene expression implies a highly complex relationship between these variants, transcription factor binding and regulation of SMAD7.

The effects of this risk haplotype on CRC development may be related to a transient cellular condition. We found that fragment A was responsive to BMP4 stimulation in a manner that exaggerated the differences between the CGTC and TCCG haplotypes. As none of the 4 SNPs disrupt a canonical BMP-response element motif, it remains an open question how this signal is transmitted. We also do not know how responsiveness in this one enhancer element interacts with other enhancer, repressor or promoter elements regulating *SMAD7*. However, the lack of responsiveness to TGFβ1, and the similar levels of activity for the two haplotypes in cell line RKO imply that the allele specific enhancer effects are in response to a specific set of cell conditions. Thus, haplotype differences could modulate SMAD7 as a negative regulator of TGFβ superfamily signaling. Further studies will be required to determine if other signaling pathways use this enhancer, and if they do so, whether those effects are in an allele-specific manner.

It is also important to note that *SMAD7* is also a regulator of TGFβ signaling in immune cells that are linked to ulcerative colitis and inflammatory bowel syndrome, both risk factors for CRC [34]–[36]. The role of the enhancer in immune cells in the subsequent relationship to CRC development is not known.

We cannot exclude the possibility that there may be additional functional SNPs within this region that we have not identified. However, fine mapping studies also identified the region containing the 4 functional SNPs as being associated with CRC risk, and rs6507874, rs8085824 and rs58920878 were ranked in the top ten SNP associations in the region [37]. We did not account for multiple testing in our eQTL analysis. However, we argue that Bonferroni testing is unduly conservative due to the fact that the SNPs in the putative risk haplotype are in very strong LD (D′ of 0.94–1, 1000 Genomes Project data). We also cannot conclude that *SMAD7* is the only target of the enhancer we describe in this study as no genome wide analyses of long-range chromatin interactions to date have been performed. This emphasizes the need for future studies such as CRISPR/Cas9 knock out of the putative enhancer region to support our hypothesis that this regulatory element modulates *SMAD7* expression.

Several studies have noted that GWAS associated risk SNPs map to enhancers, particularly super-enhancers, at higher than random rates. In this study we provide another example of multiple functional variants mapping to regulatory regions. Also, when considering colon cancer associated SNPs from GWAS studies, several are found in the vicinity of other TGFβ superfamily members. If modulation of *SMAD7* expression leads to CRC, as we propose here, it seems natural that signaling targets of SMAD7 would also be implicated in CRC risk. Identification of the functional variants tied to the regulation of those genes, including *BMP2* and *BMP4*, will clarify the role of TGFβ signaling in the etiology of CRC.

## Materials and Methods

Tissue collection was approved by the following Institutional Review Boards of the participating centers and all subjects provided written informed consent: Dartmouth College Committee for the Protection of Human Subjects, Maine Medical Center IRB, Beth Israel Deaconess Medical Center Committee on Clinical Investigations, Lahey Hospital IRB, University of Vermont IRB, University of Southern California IRB, Kaiser Permanente, Los Angeles IRB, Colorado Multiple IRB, University of Iowa IRB, University of Minnesota IRB, Minneapolis VAMC IRB, Allina Health IRB, University of Toronto IRB, Sunnybrook Hospital IRB, St. Michaels Hospital IRB, Henry Ford Hospital IRB, University of North Carolina IRB, Cleveland Clinic IRB, and Kaiser Permanente, Cleveland IRB.

### Cell Culture

HCT-116, SW480, and RKO CRC cell lines and the HEK293 cell line were obtained from the American Type Culture Collection (ATCC, Manassas, VA). HCT-116 and SW480 cells were grown in McCoy's 5A (Mediatech) supplemented with 10% Fetal Bovine Serum (Omega Scientific, Inc.), and 1% Penicillin/Streptomycin, and incubated at 37°C and 5% CO_2_. RKO and HEK293 cells were grown in DMEM (Mediatech) supplemented with 10% Fetal Bovine Serum (Omega Scientific, Inc.), and 1% Penicillin/Streptomycin, and incubated at 37°C and 5% CO_2_. Cells treated with 100 pM BMP4 (Sigma-Aldrich) or 100 pM TGFβ1 (Sigma-Aldrich) were serum starved overnight prior to performing luciferase assays or harvesting of nuclear extracts for EMSA.

### Plasmids and Luciferase Assays

DNA fragments corresponding to candidate enhancer regions were PCR amplified using genomic DNA from a normal lymphoblastoid cell line (See Supplemental Methods in File S1 for primer sequences). Fragments were amplified and cloned using CloneAmp HiFi PCR Premix and the In-Fusion HD cloning kit (Clontech). PCR fragments were subcloned into the Sac II restriction enzyme site in both directions, upstream of a thymidine kinase (TK) minimal promoter-firefly-luciferase vector obtained courtesy of Dr. G. A. Coetzee (USC). PCR fragments were sequenced in both directions followed by Sanger sequencing to confirm the presence of the candidate variants and the absence of any PCR amplification-induced mutations (Genewiz). A region of Chr 8 served as the negative control [38], and a region of 8q24 which was previously shown to have enhancer activity in HCT-116 (AcP 6) served as the positive control [38]. For enhancer assays SW480 (10×10^4^ cells/well), HCT-116, RKO, or HEK293 cells (6×10^4^ cells/well) were seeded into 96-well plates. Cells were co-transfected with reporter plasmids and constitutively active pRL-TK Renilla luciferase plasmid (Promega) using Lipofectamine 2000 Reagent (Life Technologies) according to the manufacturer's instructions. After 24 hrs cells were harvested and extracts were assayed for luciferase activity using the Dual-Luciferase Reporter Assay System (Promega) according to the manufacturer's instructions, and measured using a Tecan Infinite F200Pro Microplate Reader. The ratio of luminescence from the experimental sample to the negative control reporter was calculated for each sample, and defined as the relative luciferase activity. Luciferase activity was tested in a minimum of three independent clones for each construct. The data are presented as mean ± SD of at least three independent transfection experiments each conducted in triplicate. Relative luciferase activity greater than 2 was considered positive for activity. To assess allele-specific effects, specific SNP alleles were generated by mutagenesis using the QuikChange site-directed mutagenesis kit (Agilent Technologies). Plasmids were sequenced and transfected into the cells as above. The mutagenesis data are presented as mean fold change ± SD of at least three independent transfection experiments each conducted in triplicate. Two-side p-values between alleles were calculated using the student t-test.

### Electrophoretic Mobility Shift Assay (EMSA)

Near-infrared dye (Li-Cor Bioscience) labeled EMSA oligonucleotide probes spanning the SNPs rs6507874, rs6507875, rs8085824, and rs58920878 were synthesized by Integrated DNA Technologies and annealed in 1X TE (see Supplemental Methods in File S1 for sequences). Nuclear extracts from HCT116, SW480, and RKO CRC cell lines were prepared using the NE-PER nuclear and cytoplasmic extraction kit (Pierce, Thermo Scientific). Binding reactions containing 10X binding buffer (100 mM Tris, 500 mM KCL, 10 mM DTT, pH 7.5), 1µg poly(dI•dC), 2.5 mM DTT/0.25% Tween 20, and 5 µg nuclear extract were preincubated with 20 pmol unlabeled competitor DNA at room temperature for 10 minutes. IRDye labeled oligos were added (50 fmol per oligo) and reactions were incubated in darkness for 20 min at room temperature prior to addition of 10X Orange loading dye (Li-Cor Biosciences). Complexes were resolved on a 6% native polyacrylamide gel run at 200 V for 90 min at 4° in 0.5X TBE and imaged in the glass plates using a Li-Cor Odyssey Imager.

### DNA and RNA Isolation

RNA and DNA were extracted from 320 fresh frozen tissue biopsies of normal colorectal mucosa that were obtained from surveillance colonoscopy as part of the Aspirin/Folate Polyp Prevention Study [26], [27]. The tissue was homogenized using the Precellys Minilys bead mill (Bertin Technologies) and total RNA was extracted using the mirVana kit (Life Technologies). Genomic DNA was extracted using the MELT kit (Life Technologies). The Aspirin/Folate Polyp Prevention Study was approved by the Institutional Review Boards of the participating centers and all subjects provided written informed consent.

### Genotyping

The genotypes of rs6507874, rs6507875, rs8085824, and rs4939827 were determined using TaqMan SNP genotyping assays (Life Technologies) and the Type-it Fast SNP Probe PCR Kit (Qiagen). Assays were read using an Applied Biosystems 7900HT Real Time Instrument and analyzed with the manufacturer's software, SDS2.3 (Life Technologies).

### Expression Quantitative Trait Loci (eQTL) Analysis

Following quantitation, 308 samples had sufficient RNA to proceed to cDNA synthesis using 250 ng of total RNA with the High Capacity RNA-to-cDNA kit (Life Technologies). cDNA samples were then preamplified with TaqMan Preamp Master Mix and 96 TaqMan Gene Expression Assays (Life Technologies), and loaded on a microfluidics chamber for real time PCR analysis (96.96 Dynamic Array and BioMark HD system, Fluidigm). Relative quantity of expression of *SMAD7* (Hs00998193_m1), *CTIF* (Hs00969548_m1), and *DYM* (Hs00214264_m1) was measured using the comparative C_T_ Method (ΔΔC_T_). Expression was normalized using beta-Glucuronidase (Hs00939627_m1), determined to show stable gene expression (0.221) in normal colon tissues using NormFinder [39]. The effect of each minor allele (0, 1 or 2) on fold change (FC) was evaluated using a rank-based non-parametric analysis and assuming an additive genetic model [40]. Linear regression modeling of the SNP genotype by the rank-transformed log_2_(ΔΔCt) was adjusted for age, gender and race. Test for significance was obtained from a likelihood ratio test and a 2-sided *p*-value <0.05 was considered statistically significant. Log_2_(ΔΔCt) was plotted as a function of genotypes using a box plot – dot plot overlay. All data were analyzed with R 12.13.1 using the SNPassoc package.

## Supporting Information

Figure S1 **(A)** Fragment A does not exhibit enhancer activity in HEK293 cells. The 2 kb fragment containing either the CGTC (major) or TCCG (minor) haplotype does not demonstrate enhancer activity in the non-colon derived cell line HEK293. **(B)** Fragment A haplotypes TCTC (9.5% in CEU population) and CCTC (5.9% in CEU population) demonstrate activity levels between that of the major CGTC and TCCG haplotypes. Light bars represent cell line HCT-116 and dark bars represent SW480.
(TIF)

Figure S2 **Differential protein binding by rs6507874 alleles using EMSA.** **(A)** Nuclear extracts from SW480, HCT-116, and RKO cell lines were incubated with IR-dye labeled 33mers centered on rs6507874 C (red label) and T (green labels) prior to native EMSA as labeled. Unlabeled competitors are in 200-fold excess to labeled probes. Competitor X is an unmatching sequence with similar nucleotide content. Top panel shows the gels as a merged color image. Middle panel shows the red (700) channel image of the C probe in black and white, and bottom panel shows the green (800) channel of the T probe in black and white, for reproduction clarity. Bands specific for one allele and lost upon competition are marked with arrows.
(TIF)

Figure S3 **Differential protein binding by rs6507875 alleles using EMSA.** **(A)** Nuclear extracts from SW480, HCT-116, and RKO cell lines were incubated with IR-dye labeled 33mers centered on rs6507875 C (red label) and G (green labels) prior to native EMSA as labeled. Unlabeled competitors are in 200-fold excess to labeled probes. Competitor X is an unmatching sequence with similar nucleotide content. Top panel shows the gels as a merged color image. Middle panel shows the red (700) channel image of the C probe in black and white, and bottom panel shows the green (800) channel of the G probe in black and white, for reproduction clarity. Bands specific for one allele and lost upon competition are marked with arrows.
(TIF)

Figure S4 **Differential protein binding by rs8085824 alleles using EMSA.** **(A)** Nuclear extracts from SW480, HCT-116, and RKO cell lines were incubated with IR-dye labeled 33mers centered on rs8085824 C (red label) and T (green labels) prior to native EMSA as labeled. Unlabeled competitors are in 200-fold excess to labeled probes. Competitor X is an unmatching sequence with similar nucleotide content. Top panel shows the gels as a merged color image. Middle panel shows the red (700) channel image of the C probe in black and white, and bottom panel shows the green (800) channel of the T probe in black and white, for reproduction clarity. Bands specific for one allele and lost upon competition are marked with arrows.
(TIF)

Figure S5 **Differential protein binding by rs58920878 alleles using EMSA.** **(A)** Nuclear extracts from SW480, HCT-116, and RKO cell lines were incubated with IR-dye labeled 33mers centered on rs58920878 C (red label) and G (green labels) prior to native EMSA as labeled. Unlabeled competitors are in 200-fold excess to labeled probes. Competitor X is an unmatching sequence with similar nucleotide content. Top panel shows the gels as a merged color image. Middle panel shows the red (700) channel image of the C probe in black and white, and bottom panel shows the green (800) channel of the G probe in black and white, for reproduction clarity. Bands specific for one allele and lost upon competition are marked with arrows.
(TIF)

File S1 **Supporting Tables.** Constructs and oligonucleotides used in this study. Tables S1, S2, and S3 showing differential transcription factor binding candidates.
(PDF)
