# Supplementary figures and images for: Multiple Functional Risk Variants in a SMAD7 Enhancer Implicate a Colorectal Cancer Risk Haplotype
Source: PLoS One. 2014 Nov 6;9(11):e111914. doi: 10.1371/journal.pone.0111914 (PMC4223076; doi:10.1371/journal.pone.0111914)

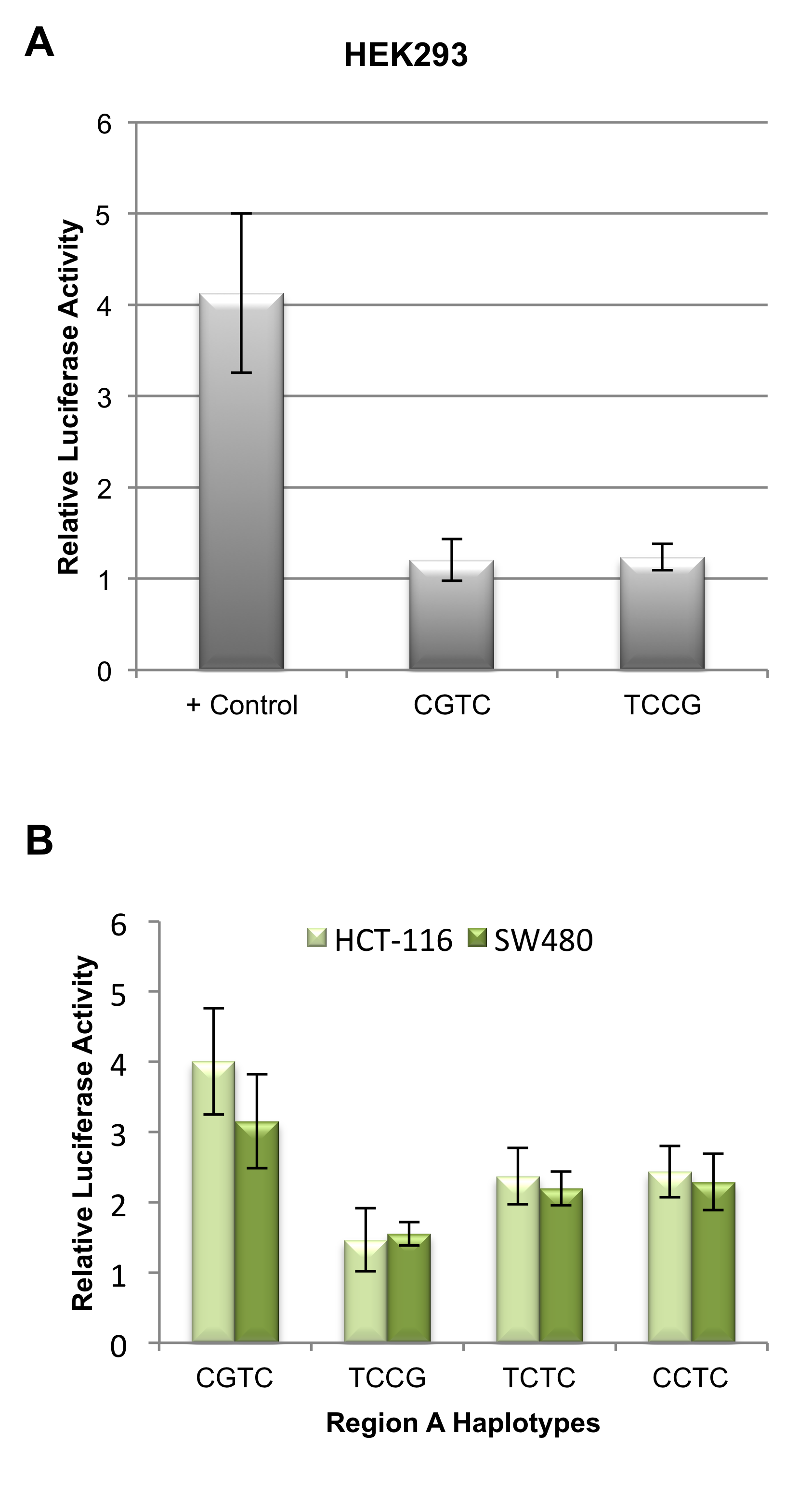

Supplement: Figure S1 — (A) Fragment A does not exhibit enhancer activity in HEK293 cells. The 2 kb fragment containing either the CGTC (major) or TCCG (minor) haplotype does not demonstrate enhancer activity in the non-colon derived cell line HEK293. (B) Fragment A haplotypes TCTC (9.5% in CEU population) and CCTC (5.9% in CEU population) demonstrate activity levels between that of the major CGTC and TCCG haplotypes. Light bars represent cell line HCT-116 and dark bars represent SW480. (TIF) [file pone.0111914.s001.tif]

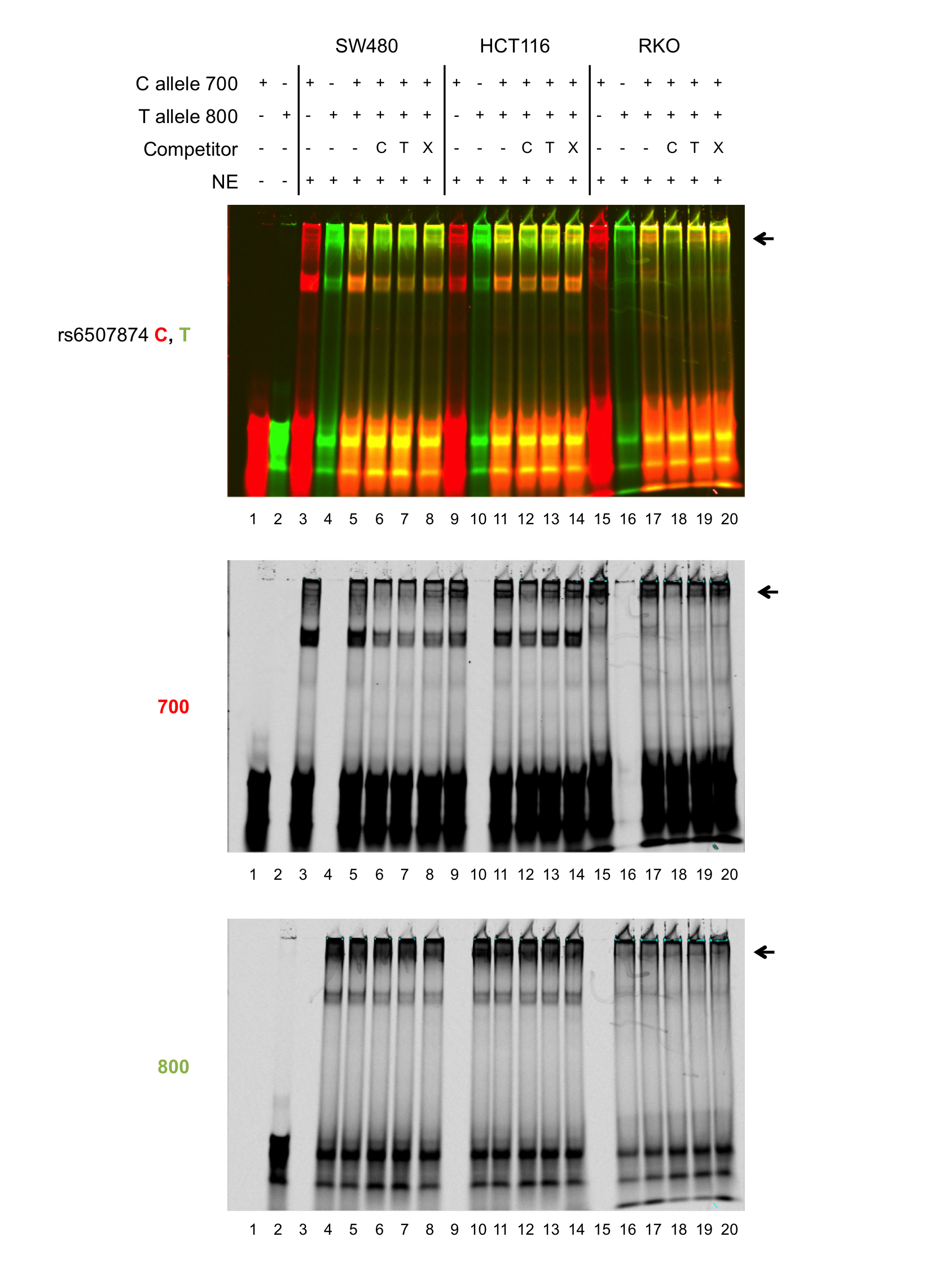

Supplement: Figure S2 — Differential protein binding by rs6507874 alleles using EMSA. (A) Nuclear extracts from SW480, HCT-116, and RKO cell lines were incubated with IR-dye labeled 33mers centered on rs6507874 C (red label) and T (green labels) prior to native EMSA as labeled. Unlabeled competitors are in 200-fold excess to labeled probes. Competitor X is an unmatching sequence with similar nucleotide content. Top panel shows the gels as a merged color image. Middle panel shows the red (700) channel image of the C probe in black and white, and bottom panel shows the green (800) channel of the T probe in black and white, for reproduction clarity. Bands specific for one allele and lost upon competition are marked with arrows. (TIF) [file pone.0111914.s002.tif]

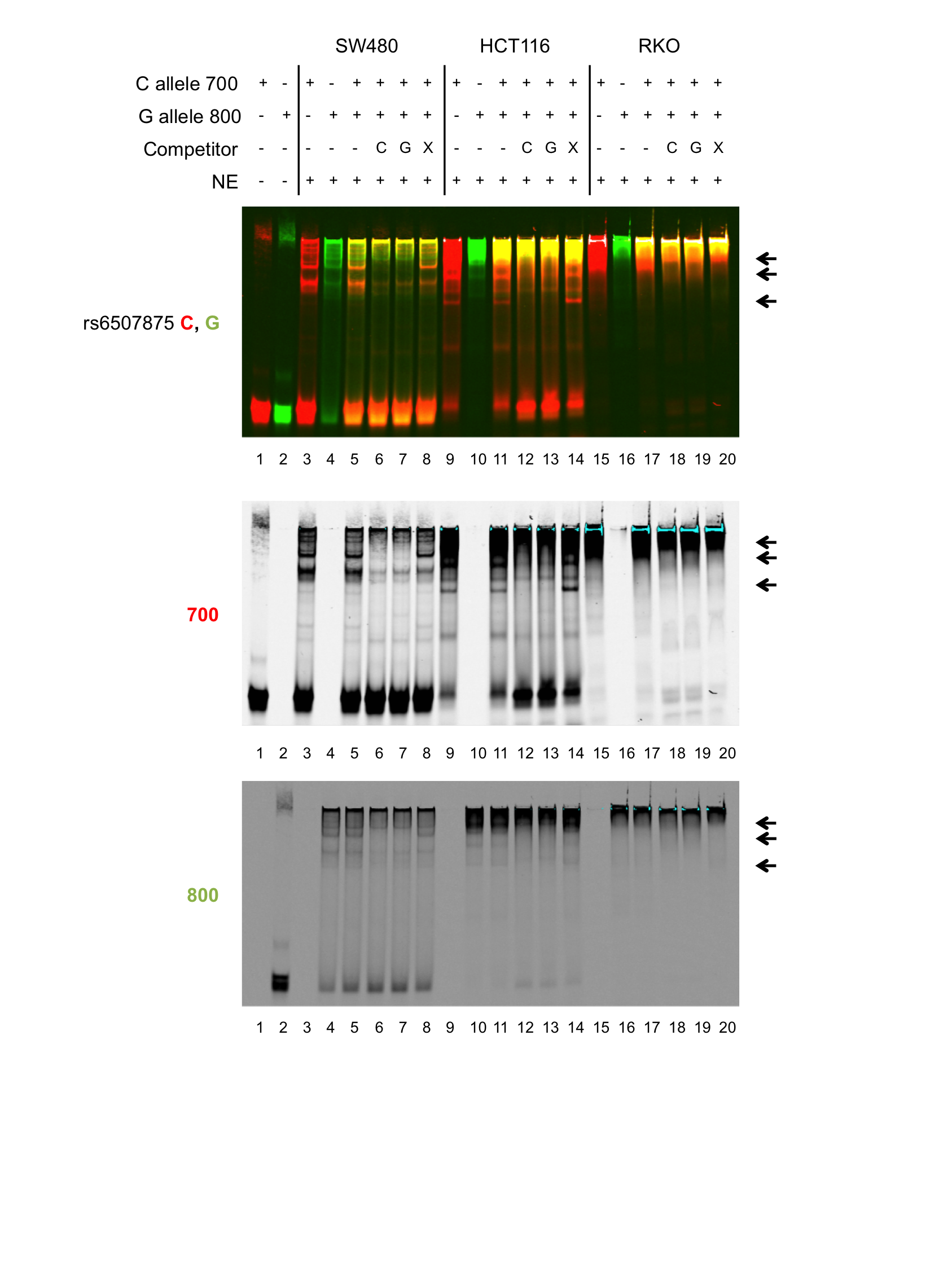

Supplement: Figure S3 — Differential protein binding by rs6507875 alleles using EMSA. (A) Nuclear extracts from SW480, HCT-116, and RKO cell lines were incubated with IR-dye labeled 33mers centered on rs6507875 C (red label) and G (green labels) prior to native EMSA as labeled. Unlabeled competitors are in 200-fold excess to labeled probes. Competitor X is an unmatching sequence with similar nucleotide content. Top panel shows the gels as a merged color image. Middle panel shows the red (700) channel image of the C probe in black and white, and bottom panel shows the green (800) channel of the G probe in black and white, for reproduction clarity. Bands specific for one allele and lost upon competition are marked with arrows. (TIF) [file pone.0111914.s003.tif]

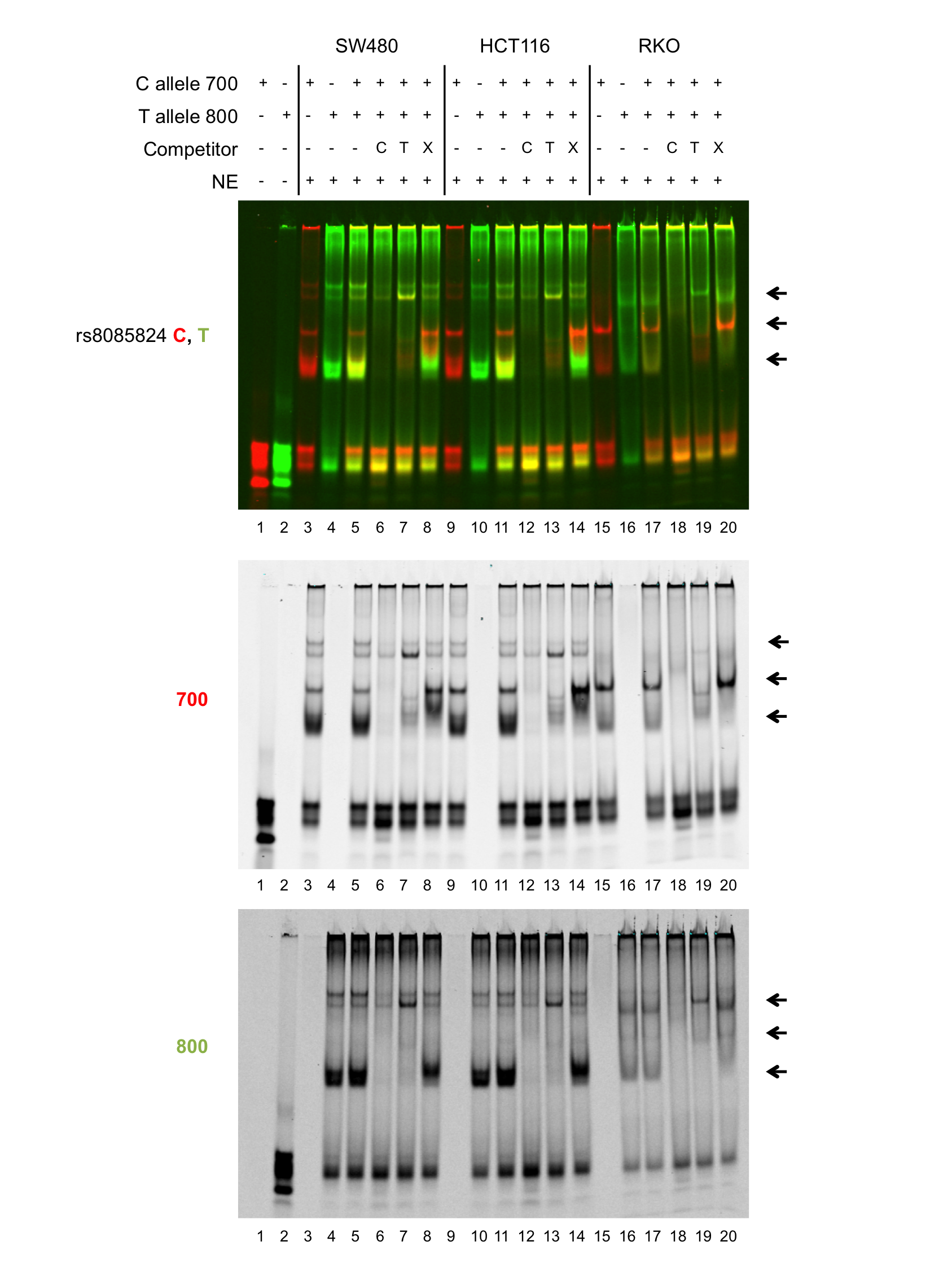

Supplement: Figure S4 — Differential protein binding by rs8085824 alleles using EMSA. (A) Nuclear extracts from SW480, HCT-116, and RKO cell lines were incubated with IR-dye labeled 33mers centered on rs8085824 C (red label) and T (green labels) prior to native EMSA as labeled. Unlabeled competitors are in 200-fold excess to labeled probes. Competitor X is an unmatching sequence with similar nucleotide content. Top panel shows the gels as a merged color image. Middle panel shows the red (700) channel image of the C probe in black and white, and bottom panel shows the green (800) channel of the T probe in black and white, for reproduction clarity. Bands specific for one allele and lost upon competition are marked with arrows. (TIF) [file pone.0111914.s004.tif]

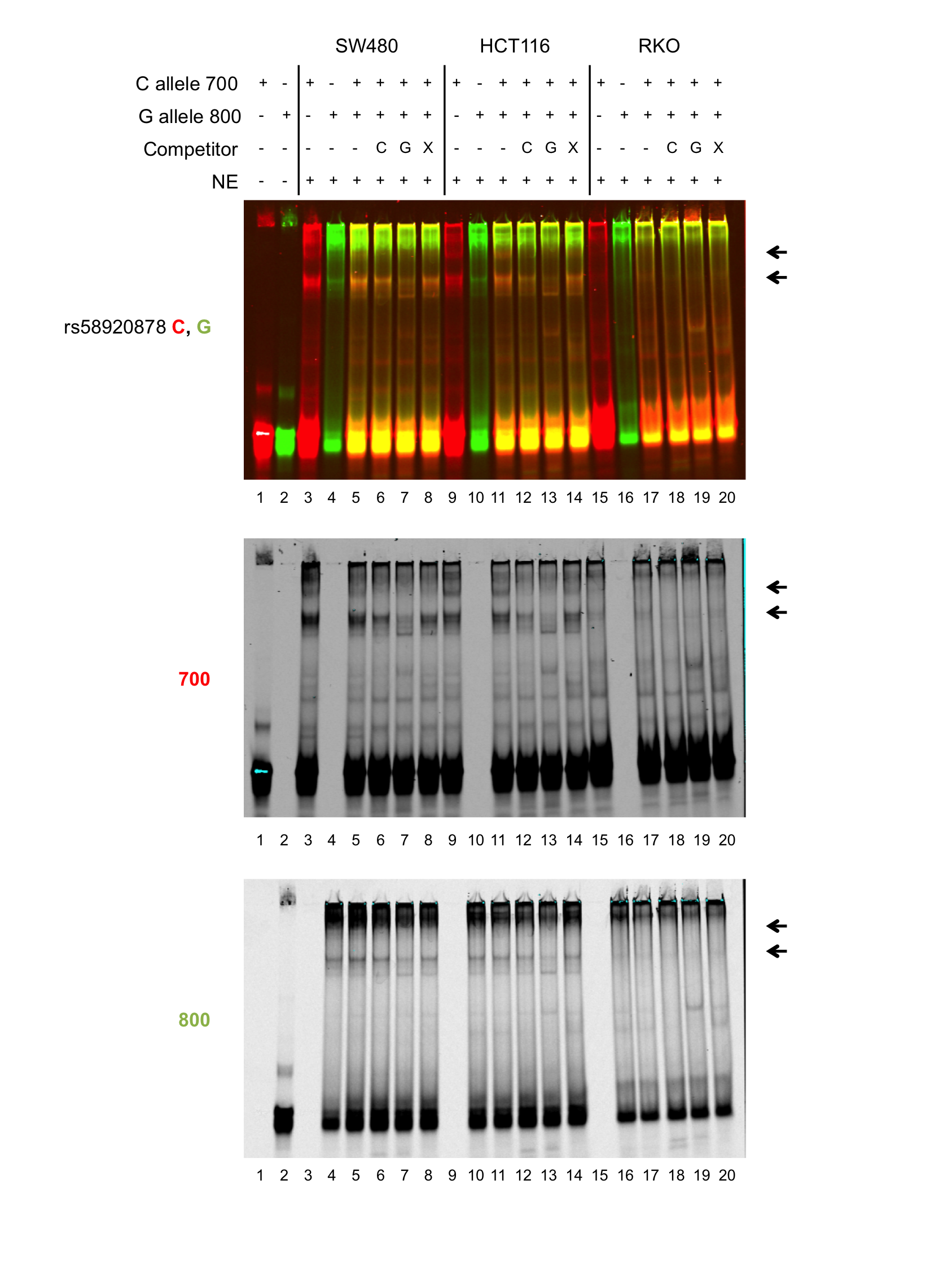

Supplement: Figure S5 — Differential protein binding by rs58920878 alleles using EMSA. (A) Nuclear extracts from SW480, HCT-116, and RKO cell lines were incubated with IR-dye labeled 33mers centered on rs58920878 C (red label) and G (green labels) prior to native EMSA as labeled. Unlabeled competitors are in 200-fold excess to labeled probes. Competitor X is an unmatching sequence with similar nucleotide content. Top panel shows the gels as a merged color image. Middle panel shows the red (700) channel image of the C probe in black and white, and bottom panel shows the green (800) channel of the G probe in black and white, for reproduction clarity. Bands specific for one allele and lost upon competition are marked with arrows. (TIF) [file pone.0111914.s005.tif]
