# Supplementary material for: Multiple Functional Risk Variants in a SMAD7 Enhancer Implicate a Colorectal Cancer Risk Haplotype
Source: PLoS One. 2014 Nov 6;9(11):e111914. doi: 10.1371/journal.pone.0111914 (PMC4223076; doi:10.1371/journal.pone.0111914)
Supplement: File S1 — Supporting Tables. Constructs and oligonucleotides used in this study. Tables S1, S2, and S3 showing differential transcription factor binding candidates. (PDF) [file pone.0111914.s006.pdf]

Supplemental Materials and Methods

Constructs used in study

Name	Genomic Coordinates of Insert	Primers	SNPs within Construct Insert
A	chr18:46448427-46450512	TAGGTACCGAGCTCCGCCAGGCTCTTACAGCCCTTTG, TCTCAGCTGCTCGAGCCGATGCCAATGTGTCCTTCCT	rs6507874, rs6507875, rs8085824, rs58920878
A1	chr18:46448646-46449078	TAGGTACCGAGCTCCGCTTTCAACTGGATGGAACCTTG, TCTCAGCTGCTCGAGCCAATCTTCTGTCTGGGCTGGA	rs6507874, rs6507875
A2	chr18:46449057-46449506	TAGGTACCGAGCTCCGCCCAGACAGAAGATTATGTGTCTCAA, TCTCAGCTGCTCGAGCCTACAAGACTTCCGGTCTCCA	rs8085824
A3	chr18:46449051-46449664	TAGGTACCGAGCTCCGCTCCAGCCCAGACAGAAGATT, TCTCAGCTGCTCGAGCCAAGGCCACACCAAAACACAT	rs8085824, rs58920878
A4	chr18:46449427-46449942	TAGGTACCGAGCTCCGCCAGACTGCAGGAACGCTGT, TCTCAGCTGCTCGAGCCGCAGTGTGCACATTTGTATGG	rs58920878
B	chr18:46450925-46451320	TAGGTACCGAGCTCCGCAGCCCAGAAAAACTGCAATG, TCTCAGCTGCTCGAGCCGGGAATTCAGGGCAAAGG	rs12956924, rs34007497
C	chr18:46451457-46452057	TAGGTACCGAGCTCCGCCCTCTGAAGGCAAAACTCACA, TCTCAGCTGCTCGAGCCCACCCTGTGCCATTGAGAC	rs4939567
D	chr18:46453028-46453391	TAGGTACCGAGCTCCGCCTTGCACCTGGCAACCTG, TCTCAGCTGCTCGAGCCTCTATTTGGGGGAGGGATTC	rs11874392
E	chr18:46453066-46453854	TAGGTACCGAGCTCCGCAGGGTCCTCCCTCCTCAG, TCTCAGCTGCTCGAGCCCCATGGCTAGAATGCGCTAA	rs11874392, rs4939827, rs35352860
F	chr18:46453693-46454148	TAGGTACCGAGCTCCGCCTTTGCAGCATCTCCAGTGA, TCTCAGCTGCTCGAGCCGGGATGTGGAGACAATCAGG	rs35352860, rs12953717, rs7226855
G	chr18:46458022-46459036	TAGGTACCGAGCTCCGCGATCACCGAAGGGACTTGG, TCTCAGCTGCTCGAGCCTCACATTCATCCTTCTCTGTCTG	rs9946510, rs6507877, rs4464148

Oligonucleotides used in this study

Name	Label	Sequence
rs6507874 C allele		CCA GGT GTC TAG ACT CCG GGA CCA CCT ATG GGA
rs6507874 T allele		CCA GGT GTC TAG ACT CTG GGA CCA CCT ATG GGA
rs6507875 C allele		TCC GGG ACC ACC TAT GCG ATG GTC TCT GGG TGC
rs6507875 G allele		TCC GGG ACC ACC TAT GGG ATG GTC TCT GGG TGC
rs8085824 C allele		CCC CCT TCT CCC CTT CCC TGC CGG CAC CCC CAT
rs8085824 T allele		CCC CCT TCT CCC CTT CTC TGC CGG CAC CCC CAT
rs58920878 C allele		GTC AGG GCC TTG CCC TCG CTC CCT GCA GCC CCA
rs58920878 G allele		GTC AGG GCC TTG CCC TGG CTC CCT GCA GCC CCA
rs6507874 C allele F	700	/5IRD700/CCA GGT GTC TAG ACT CCG GGA CCA CCT ATG GGA
rs6507874 C allele R	700	/5IRD700/TCC CAT AGG TGG TCC CGG AGT CTA GAC ACC TGG
rs6507874 T allele F	800	/5IRD800/CCA GGT GTC TAG ACT CTG GGA CCA CCT ATG GGA
rs6507874 T allele R	800	/5IRD800/TCC CAT AGG TGG TCC CAG AGT CTA GAC ACC TGG
rs6507875 C allele F	700	/5IRD700/TCC GGG ACC ACC TAT GCG ATG GTC TCT GGG TGC
rs6507875 C allele R	700	/5IRD700/GCA CCC AGA GAC CAT CGC ATA GGT GGT CCC GGA
rs6507875 G allele F	800	/5IRD800/TCC GGG ACC ACC TAT GGG ATG GTC TCT GGG TGC
rs6507875 G allele R	800	/5IRD800/GCA CCC AGA GAC CAT CCC ATA GGT GGT CCC GGA
rs8085824 C allele F	700	/5IRD700/CCC CCT TCT CCC CTT CCC TGC CGG CAC CCC CAT
rs8085824 C allele R	700	/5IRD700/ATG GGG GTG CCG GCA GGG AAG GGG AGA AGG GGG
rs8085824 T allele R	800	/5IRD800/ATG GGG GTG CCG GCA GAG AAG GGG AGA AGG GGG
rs8085824 T allele F	800	/5IRD800/CCC CCT TCT CCC CTT CTC TGC CGG CAC CCC CAT
rs58920878 C allele F	700	/5IRD700/GTC AGG GCC TTG CCC TCG CTC CCT GCA GCC CCA
rs58920878 C allele R	700	/5IRD700/TGG GGC TGC AGG GAG CGA GGG CAA GGC CCT GAC
rs58920878 G allele R	800	/5IRD800/TGG GGC TGC AGG GAG CCA GGG CAA GGC CCT GAC
rs58920878 G allele F	800	/5IRD800/GTC AGG GCC TTG CCC TGG CTC CCT GCA GCC CCA
Competitor X		GAG CCA CCG AGC CTG CAC CAC CAA GGG

Transcription Factor	Position	Motif sequence with rs6507874 and/or rs6507875 denoted	CG haplotype motif scores		Change with TC alleles	
			Core	Matrix	Δ Core	Δ Matrix
MECP2	14 (-)	tagacTC(C/T)GG	1.000	0.925	0.245	0.199
RBP-Jkappa	29 (+)	cctaTG(G/C)GAtg	1.000	0.704	0.102	0.070
RBP-Jkappa	17 (+)	actc(C/T)GGGAcc	0.745	0.545	-0.255	-0.174
Churchill	21 (+)	(C/T)GGGAc	0.998	0.991	0.014	0.014
YY1-like	29 (-)	ccTATG(G/C)	0.996	0.938	0.200	0.189
CTCF	21 (-)	(c/t)gggaCCACCtatg(g/c)gatgg	0.959	0.822	0.000	0.136
muscle	28 (-)	acctatg(G/C)GATGgtctctggg	0.932	0.836	0.052	0.036
AIRE	14 (-)	tagactc(c/t)gggacCACCTatg(g/c)gatg	0.925	0.862	0.000	0.180
hltf	28 (-)	acCTATG(g/c)ga	0.921	0.925	0.000	0.095
muscle	18 (+)	ctc(c/t)gggacCACCTatg(g/c)gat	0.912	0.839	0.000	0.017
KLF4	33 (-)	tg(G/C)GATG	0.906	0.910	0.246	0.234
Ikaros	19 (+)	tc(c/t)GGGACca	0.903	0.802	0.000	-0.064
Tef-1-related	32 (+)	atG(G/C)GATgg	0.900	0.824	0.128	0.102
Pax	20 (+)	C(C/T)GGGaccacc	0.735	0.670	-0.165	-0.088
Ikaros	31 (+)	tatG(G/C)GATgg	0.897	0.887	0.269	0.226
NF-1A	20 (+)	C(C/T)GGGa	0.714	0.716	-0.170	-0.168
NF-1A	32 (+)	ATG(G/C)Ga	0.873	0.874	0.307	0.305
BEN	15 (-)	agaCTC(C/T)G	0.717	0.652	-0.123	-0.113
SREBP	30 (-)	ctatg(g/c)GATGGtctc	0.830	0.838	0.000	0.045
DBP	35 (-)	(g/c)gATGGT	0.788	0.765	0.000	0.041
RFX-related	7 (-)	agGTGTCtagactc(c/t)gg	0.786	0.740	0.000	0.040
Sp100	27 (-)	cacctatg(G/C)GATGgt	0.477	0.499	-0.269	-0.225
GR-like	21 (-)	(c/t)GGGACcacctatg(g/c)g	0.741	0.606	0.000	-0.044
BEN	20 (+)	C(C/T)GGGacc	0.725	0.750	-0.012	-0.011
Zic	13 (-)	ctagaCTC(C/T)Gggacc	0.524	0.492	-0.196	-0.128
AP-2	25 (+)	acCACCTatg(g/c)gatg	0.719	0.643	0.000	0.136
HOMEZ	27 (-)	cacctaTG(G/C)GAtggtct	0.471	0.577	-0.241	-0.166
CDP	27 (+)	cACCTAtg(g/c)g	0.711	0.772	0.000	0.055
HOMEZ	28 (+)	acctatG(G/C)GATggtctc	0.451	0.383	-0.227	-0.157
Rhox11	10 (-)	tgtctagACTC(C/T)gggac	0.669	0.592	0.185	0.143
HDX	30 (-)	ctatg(G/C)GATGgtctctg	0.666	0.612	0.007	0.005
Rhox11	31 (+)	tatg(g/c)GATGGtctctgg	0.661	0.583	0.000	-0.019
HD-SINE	30 (-)	ctatg(G/C)GATGgtctctg	0.656	0.553	-0.004	-0.003
HDX	10 (-)	tgtctAGACTc(c/t)gggac	0.614	0.586	0.000	0.061
AP-2	25 (-)	accacctaTG(G/C)GAtg	0.574	0.643	0.281	0.136
Rhox11	14 (+)	tagacTC(C/T)GGgaccacc	0.324	0.370	-0.243	-0.188
HD-SINE	16 (-)	gactc(C/T)GGGAccaccta	0.408	0.494	-0.149	-0.109
Hox9-12	26 (-)	ccaccTATG(G/C)gatggtc	0.398	0.527	-0.152	-0.095
Hox-13	27 (-)	caccTATG(G/C)gatggtc	0.458	0.540	-0.079	-0.048
Cphx	20 (+)	c(c/t)gggacCACCTat	0.510	0.396	0.000	-0.129

Table S1. Differential Transcription Factor Binding Candidates for rs6507874 and rs6507875. Biobase Match search using Matrix library: TRANSFAC MATRIX TABLE, Release 2013.4, and Profile: vertebrate_non_redundant.prf, minimize false negatives. Candidate TF list was determined using the CG haplotype +/- 20 bp from each SNP inclusive of the 13 bp between the two SNPs. Predicted TFs with a change in core and matrix scores < 0.010 upon search using the TC haplotype were eliminated. Differences in core and matrix scores with the TC haplotype are listed in the right two columns. Proteins predicted to preferentially bind the CG haplotype are highlighted in red; proteins predicted to preferentially bind the TC haplotype are highlighted in green. Darker shades indicate greater differences in core and matrix scores. SNP position is underlined in binding sequence with rs6507874 in red and rs6507875 in blue. Position and orientation of motif start site relative to the beginning of the 55 mer search sequence are listed in column 2.

Transcription Factor	Position	Motif sequence with rs8085824 denoted	T allele motif scores		Change with C allele	
			Core	Matrix	Δ Core	Δ Matrix
RUSH-1alpha	14 (+)	cccCTTC(T/C)ct	0.989	0.986	0.124	0.111
Blimp-1	15 (-)	cccTTC(T/C)Ctgccgg	0.949	0.826	-0.037	-0.022
Sp100	18 (-)	ttc(t/c)ctgcCGGCAcc	0.862	0.765	0.000	0.038
Ikaros	20 (-)	c(t/c)CTGCCggc	0.832	0.760	0.000	-0.024
YY1	16 (+)	CCTTC(t/c)c	0.813	0.806	0.000	0.035
Zic1	18 (-)	ttc(t/c)cTGCCGgcacc	0.804	0.596	0.000	-0.150
Kaiso	19 (+)	tc(T/C)CTGCcgg	0.800	0.638	-0.200	-0.155
Ets	16 (+)	cCTTC(T/C)ct	0.783	0.730	-0.217	-0.200
Pax	18 (-)	ttc(t/c)ctGCCGG	0.718	0.598	0.000	0.022
GKLF	17 (+)	CTTC(T/C)ct	0.709	0.725	-0.233	-0.220
Churchill	18 (-)	tTC(T/C)CT	0.656	0.657	-0.327	-0.323
Helios A	1 (-)	cccTTC(T/C)Ctgc	0.633	0.489	-0.309	-0.232
Ikaros	16 (-)	ccTTC(T/C)Ctgc	0.628	0.613	-0.269	-0.226
LXR, PXR, CAR, COUP, RA	18 (+)	ttc(t/c)ctgccgGCACCcc	0.627	0.636	0.000	-0.059
Zic1	13 (-)	tccccTTC(T/C)Ctgccg	0.559	0.705	0.195	0.128

Table S2. Differential Transcription Factor Binding Candidates for rs8085824. Biobase Match search using Matrix library: TRANSFAC MATRIX TABLE, Release 2013.4, and Profile: vertebrate_non_redundant.prf, minimize false negatives. Candidate TF list was determined using the T allele +/- 20 bp. Predicted TFs with a change in core and matrix scores < 0.010 upon search using the C allele were eliminated. Differences in core and matrix scores with the C allele are listed in the right two columns. Proteins predicted to preferentially bind the T allele are highlighted in red; proteins predicted to preferentially bind the C allele are highlighted in green. Darker shades indicate greater differences in core and matrix scores. SNP position is underlined in binding sequence. Position and orientation of motif start site relative to the beginning of the 41mer search sequence are listed in column 2.

Transcription Factor	Position	Motif sequence with rs58920878 denoted	C allele motif scores		Change with G allele	
			Core	Matrix	Δ Core	Δ Matrix
MAZ	21 (-)	**(c/g)**gCTCCCt	1.000	0.913	0.000	0.177
SZF1-1	16 (-)	gccct**(c/g)**gctCCCTGc	1.000	0.725	0.000	-0.027
C/EBP	13 (+)	cTTGCCct**(c/g)**gct	0.995	0.831	0.000	-0.010
Pax-5	11 (-)	gccttgccct**(C/G)**GCTCcctgcagccccag	0.973	0.599	0.262	0.110
CACD	20 (+)	t**(c/g)**gCTCCC	0.948	0.772	0.000	0.117
Zic3	20 (-)	t**(c/g)**gCTCCCt	0.948	0.796	0.000	-0.013
KROX	13 (+)	cttgCCCT**(C/G)**gctcc	0.933	0.857	0.249	0.169
ZF5	15 (+)	tgccct**(C/G)**GCTCcc	0.919	0.693	0.334	0.226
MTF-1	13 (+)	ctTGCCCt**(c/g)**gctcc	0.900	0.799	0.000	0.138
SZF1-1	8 (-)	agggccttgCCCT**(C/G)**g	0.896	0.696	-0.104	-0.059
MOVO-B	17 (-)	CCCT**(C/G)**gc	0.883	0.888	0.243	0.232
TFII-I	18 (-)	CCT**(C/G)**Gctcc	0.866	0.909	0.081	0.055
ZF5	13 (+)	cttgccCT**(C/G)**GCtc	0.864	0.772	0.111	0.076
Spz1	15 (-)	tgccct**(c/g)**GCTCCctg	0.828	0.789	0.000	0.052
MAZ	17 (-)	ccCT**(C/G)**GCt	0.819	0.841	0.165	0.117
Pbx	14 (-)	ttgccct**(C/G)**GCTC	0.800	0.817	0.223	0.140
AhR	16 (-)	gccCT**(C/G)**GCtcc	0.800	0.771	0.200	0.188
GABP	19 (-)	ct**(c/g)**gCTCCCtgc	0.800	0.771	0.000	0.099
MAF	17 (-)	ccct**(c/g)**gCTCCCtgcag	0.800	0.596	0.000	0.023
MAF	7 (-)	cagggcCTTGCcct**(c/g)**g	0.800	0.547	0.000	0.010
Spz1	11 (-)	gccttgcCCT**(C/G)**Gctc	0.799	0.779	0.273	0.222
PPARgamma:RXRalpha	4 (-)	ggtcagggcctTGCCCt**(c/g)**gct	0.792	0.593	0.000	0.017
MOVO-B	19 (-)	CT**(C/G)**GCtc	0.789	0.795	0.261	0.249
c-Ets-1	16 (-)	gccct**(c/g)**gcTCCCTgc	0.773	0.764	0.000	0.012
Msx-1	21 (+)	**(c/g)**gcTCCCTg	0.766	0.806	0.000	0.076
Pax	19 (-)	ct**(c/g)**gctCCCTG	0.757	0.579	0.000	-0.030
PPARalpha:RXRalpha	8 (-)	agggCCTTGccct**(c/g)**gctccc	0.751	0.646	0.000	-0.015
CP2/LBP-1c/LSF	18 (+)	CCT**(C/G)**Gctccctgcag	0.716	0.659	-0.225	-0.141
LF-A1	19 (-)	ct**(c/g)**GCTCC	0.689	0.599	0.000	-0.181
myogenin / NF-1	7 (+)	cagggccttgccCT**(C/G)**GCtccctgcagccc	0.687	0.576	-0.213	-0.074
CACD	18 (+)	cct**(C/G)**GCTC	0.667	0.710	0.249	0.169
NF-1	17 (+)	ccCT**(C/G)**GCtccctgcagcc	0.661	0.649	-0.260	-0.246
p53	10 (+)	ggCCTTGccct**(c/g)**gctccctg	0.645	0.519	0.000	-0.063
Pax	13 (+)	CTTGCcct**(c/g)**gc	0.643	0.638	0.000	0.029
PPARalpha:RXRalpha	21 (-)	**(c/g)**gctccctgcaGCCCCagc	0.623	0.632	0.000	0.026
Pax-5	5 (-)	gtcagggcctTGCCCt**(c/g)**gctccctgcag	0.611	0.583	0.000	-0.014
Hand1:E47	11 (+)	gccttgcCCT**(C/G)**Gctcc	0.608	0.558	-0.260	-0.220
LXR, PXR, CAR, COUP, RA	20 (-)	t**(c/g)**GCTCCctgcagcccc	0.593	0.641	0.000	-0.036
SMAD	19 (-)	ct**(C/G)**GCTCcctg	0.511	0.532	-0.251	-0.232
Pax-6	14 (+)	ttgcccT**(C/G)**GCTccc	0.431	0.586	-0.198	-0.088
LF-A1	20 (+)	T**(C/G)**GCTccc	0.373	0.445	-0.306	-0.217

Table S3. Differential Transcription Factor Binding Candidates for rs58920878. Biobase Match search using Matrix library: TRANSFAC MATRIX TABLE, Release 2013.4, and Profile: vertebrate_non_redundant.prf, minimize false negatives. Candidate TF list was determined using the C allele +/- 20 bp. Predicted TFs with a change in core and matrix scores < 0.010 upon search using the G allele were eliminated. Differences in core and matrix scores with the G allele are listed in the right two columns. Proteins predicted to preferentially bind the C allele are highlighted in red; proteins predicted to preferentially bind the G allele are highlighted in green. Darker shades indicate greater differences in core and matrix scores. SNP position is underlined in binding sequence. Position and orientation of motif start site relative to the beginning of the 41mer search sequence are listed in column 2.
